# The Legacy of Egas Moniz: Triumphs and Controversies in Medical Innovation

**DOI:** 10.7759/cureus.72056

**Published:** 2024-10-21

**Authors:** Sanwal S Mehta, Shruti Vadali, Jivtesh Singh, Sukhmani K Sadana, Avi Singh

**Affiliations:** 1 Critical Care Medicine, Jackson-Madison County General Hospital, Jackson, USA; 2 Internal Medicine, Maimonides Medical Center, Brooklyn, USA; 3 Internal Medicine, Gomel State Medical University, Gomel, BLR; 4 Internal Medicine, All India Institute of Medical Sciences, Raipur, Raipur, IND; 5 Child and Adolescent Psychiatry, Jackson-Madison County General Hospital, Jackson, USA; 6 Neurology, Emory University, Atlanta, USA

**Keywords:** cerebral angiography, egas moniz, ethics in medical practice, lobotomy, nobel prize, prefrontal leucotomy

## Abstract

António Egas Moniz, a pioneering Portuguese neurologist, is renowned for developing cerebral angiography and introducing the prefrontal leucotomy (lobotomy), for which he received the Nobel Prize in 1949. Initially hailed as a breakthrough for conditions like schizophrenia and severe anxiety, lobotomy later became controversial due to severe side effects, including irreversible personality changes. The advent of neuroleptics, such as chlorpromazine, in 1952 led to the decline of lobotomy in favor of more effective and humane treatments. Moniz's work raised significant ethical concerns, particularly regarding informed consent and the procedure's long-term impact, underscoring the need for continuous advancement and ethical considerations in psychiatric care. We present a narrative review of Egas Moniz's life and his pioneering discovery of cerebral angiography, which had a significant impact and a lasting legacy in neurology. Additionally, we highlight the evolution of alternative brain surgeries that arose from modifications of his original prefrontal leucotomy.

## Introduction and background

António Caetano de Abreu Freire Egas Moniz, a Portuguese neurologist and Nobel Prize laureate, is regarded as a pioneer of contemporary psychosurgery [1]. He is known for his contributions, which include the development of cerebral angiography, a method for visualizing brain blood vessels by injecting radio-opaque substances into the carotid artery. This method is useful for diagnosing intracranial diseases. Additionally, he introduced prefrontal leucotomy, a procedure that later came to be known as lobotomy [2]. In 1949, Moniz received the Nobel Prize for this procedure, which involved using an instrument to make incisions in the prefrontal cortex that destroyed connections between the prefrontal cortex and other parts of the brain. Initially used to treat affective disorders such as severe anxiety, depression, and obsessive-compulsive disorder (OCD), the procedure was also widely applied in early treatments for schizophrenia [3]. Due to the lack of effective alternative treatment for schizophrenia, leucotomy gained global acceptance. It was believed to make life more bearable for patients and their families. Lobotomies became more common during the 1940s and 1950s, but their use decreased with the introduction of chlorpromazine [2,3]. Lobotomies faced severe criticism due to their questionable medical efficacy, lack of long-term follow-up, and the ethical dilemmas surrounding informed consent, particularly for patients with severe mental illness [3]. This narrative review delves into the life and the lasting yet controversial impact of the work of Egas Moniz.

## Review

Life of Egas Moniz

Early Life

**Figure 1:**
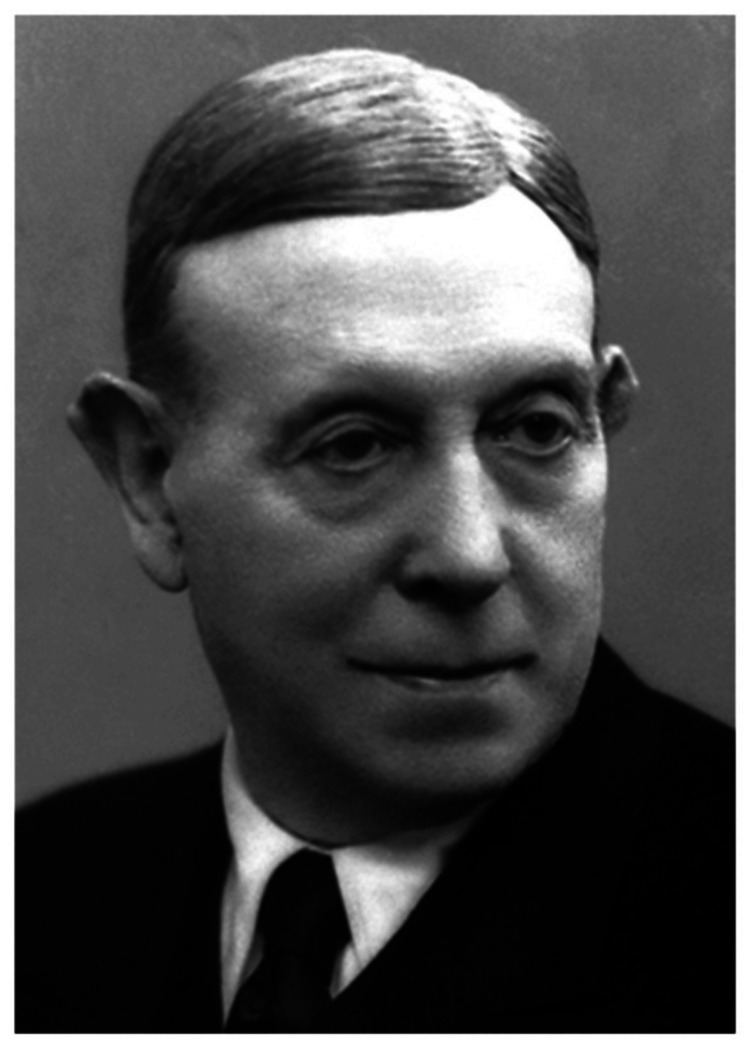
Image of António Caetano de Abreu Freire Egas Moniz Source: [4]

António Caetano de Abreu Freire Egas Moniz (Figure 1) was born in Avanca, Portugal, on November 29, 1874. He received his early education from his uncle, Abbé Caetano de Pina Rezende Abreu Sa Freire, before pursuing further studies in Bordeaux and Paris [1]. In 1902, he became a professor at the Faculty of Medicine at Coimbra University, Coimbra, Portugal. In 1911, he transferred to the newly established Chair in Neurology at Lisbon, where he remained for the rest of his career. He also worked as a physician at the Hospital of Santa Maria in Lisbon [1].

Political Career

Moniz entered politics in 1903, serving as a Deputy in the Portuguese Parliament until 1917, when he became the Portuguese Ambassador to Spain. Later that year, he was appointed Minister for Foreign Affairs and led the Portuguese delegation at the Paris Peace Conference in 1918. It was not until Moniz retired from politics in 1926, at the age of 51, that he could fully focus on his research in neurology [1].

Personal Life

Tragically, he was shot in the leg by a patient, which left him wheelchair-bound for the rest of his life [5]. Moniz, who also suffered from severe gouty arthritis in his hands, was unable to perform the leucotomies himself due to his condition and lack of neurosurgical training. He died from an internal hemorrhage on December 13, 1955, at the age of 81 [6].

 Contributions to medicine

 *Discovery of Cerebral Angiography*

The introduction of cerebral angiography, a technique still widely used today, is among his most significant contributions, though it garnered less recognition compared to his work in psychosurgery. Moniz aimed to improve the localization of brain tumors through radiographic imaging of blood vessels of the brain. He initially experimented with injecting radiopaque dyes such as strontium and lithium bromide into the brain arteries of patients with suspected brain tumors, epilepsy, and parkinsonism [5].

The initial attempts at the procedure involved a percutaneous puncture of the internal carotid artery in four patients. Only one resulted in partial film formation, while another suffered carotid rupture with contrast spilling into the subcutaneous tissue. Moniz attributed this complication to the slippage of the thin needles during the procedure. In a subsequent series, the carotid artery was surgically exposed and partially occluded in its proximal segment. Unfortunately, this led to the patient's death from a stroke eight hours later. Moniz ultimately concluded that sodium bromide was too dense to permit normal physiological blood flow [5].

**Figure 2:**
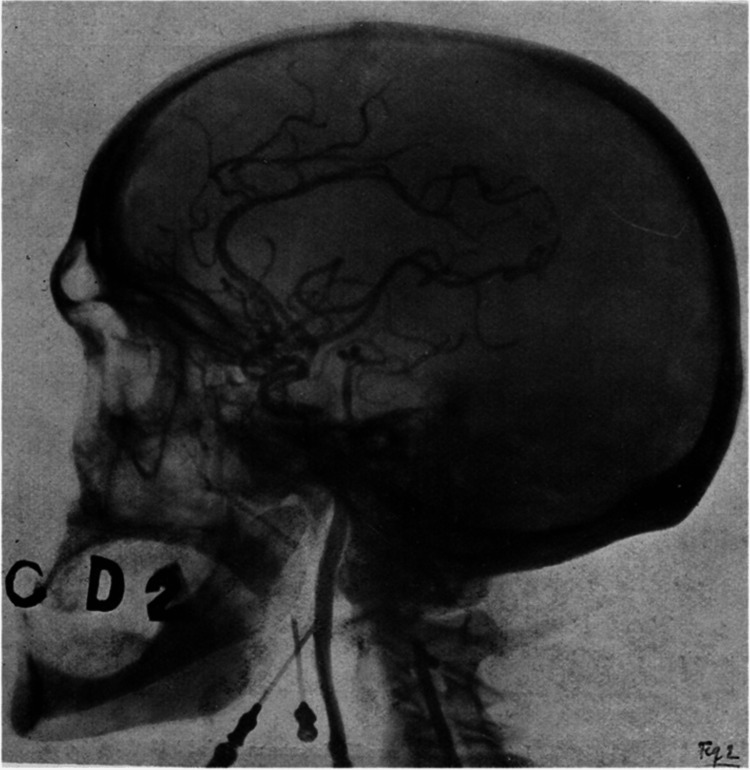
The arterial network arising from the internal carotid artery, as visualized following the injection of 30% sodium iodide in a formalin-fixed head (one of the first angiograms performed by Dr. Moniz) Source: [5]. Published under Creative Commons Attribution (https://creativecommons.org/licenses/by/4.0/) and with permission from Frontiers

Despite these setbacks, Moniz refined his approach using a 30% sodium iodide solution, which led to the first clear brain angiograms (Figure 2). Egas Moniz paid close attention to cerebrovascular anatomy and is credited with naming both the arteries of the Sylvian fissure and the carotid siphon [7].

He eventually replaced the iodine solution with thorium dioxide (also known as Thorotrast), which became his preferred contrast agent until it was discovered to be highly carcinogenic, drastically increasing the risk of leukemia and liver cancer in the 1950s [8]. Initially intended to demonstrate displacement in the vasculature, cerebral angiography replaced two previously used techniques, namely the ventriculogram and the pneumoencephalogram [9].

Egas Moniz was the first to identify the angiographic characteristics of angiomas, meningiomas, and specific vascular gliomas. He also observed the notable lack of vascularity in cysts, abscesses, and cholesteatomas. According to him, the angiographic images of most glioblastomas revealed increased vascularity around the tumor's margins, along with irregular dilations and vascular loops within the tumor and sinuses [10].

In July 1927, he presented his successful findings to the Neurological Society in Paris and the French Academy of Medicine. Moniz's work advanced the technique for visualizing the brain and improved the diagnosis of internal carotid artery occlusion, a previously missed condition [6]. The delicate procedures were performed with the help of Almeida Lima, a young neurosurgeon, as Moniz was unable to do so himself due to severe gouty arthritis [6]. Although he was nominated twice for the Nobel Prize for his cerebral imaging research, Moniz ultimately won the prize for his work in psychosurgery, leaving a lasting impact on medical science in both fields [6].

 *Discovery of Prefrontal Leucotomy*

Therapeutic methods in psychiatry developed later than in other medical fields. Early treatments for violent or dangerous patients included physical restraints, prolonged baths, and heavy sedation with opiates or barbiturates, but these had limited long-term effects [3]. In the 1930s, Manfred Sakel introduced insulin-induced hypoglycemic coma as a treatment for schizophrenia, while Ladislav von Meduna began using cardiazol for seizure therapy [11]. However, in 1938, Cerletti and Bini introduced electroconvulsive therapy, which quickly became the preferred treatment for severe depressive states and, eventually, for schizophrenia as well [3].

The origins of surgical methods to address neuropsychiatric disorders can be traced all the way back to Swiss psychiatrist Gottlieb Burckhardt in 1881, who performed surgery on six patients with hallucinations and behavioral symptoms. The results were partial, with some degree of improvement but with significant complications [12]. Given the incomplete nature of the results, Burckhardt was shunned by the medical community, leading to a period of long dormancy in the field of surgical psychiatry until 1935. In 1935, Fulton and Jacobsen performed experiments on two primates suffering from neurotic behaviors and underwent surgical separation of frontal lobes, leading to marked improvements in their symptoms [13].

**Figure 3:**
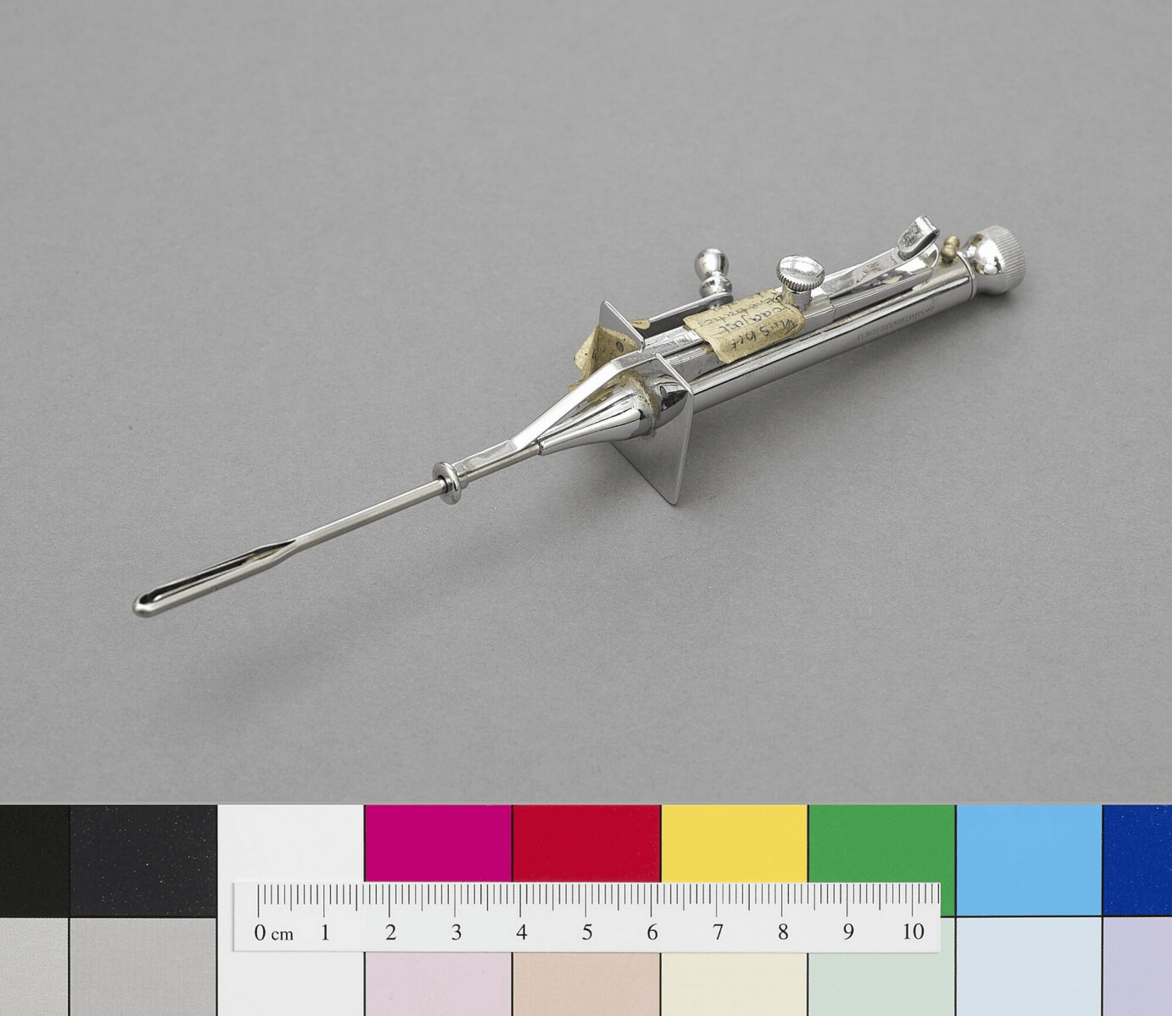
A modified version of the original leucotome, used to perform prefrontal leucotomy Source: [14]. Published under Creative Commons Attribution (https://creativecommons.org/licenses/by/4.0/legalcode)

Egas Moniz expanded on the ideas of Fulton and Jacobson and noted that certain psychoses, like schizophrenia and severe paranoia, involve persistent thought patterns that dominate normal psychological processes. He proposed that severing the nerve fibers connecting the frontal lobes with the thalamus could help realign these disruptive thought patterns. In 1936, Moniz and Almeida Lima performed the prefrontal leucotomy (later known as lobotomy), initially using alcohol to destroy the white matter in the frontal lobes [13]. Moniz then refined his technique with a leucotome, which allowed him to cut six cores in the white matter of each hemisphere (Figure 3). Early results were somewhat promising, with a few patients significantly improving [13].

However, prefrontal leucotomy had severe side effects, including early changes in behavior and personality, mental confusion, paresthesia, and pruritus, along with late sequelae like seizures (23%) and chronic headaches (15%) and a mortality rate reaching 5% [15]. Moniz emphasized that it should only be used as a last resort after other treatments had failed. The procedure became more common in the 1940s and 1950s but eventually declined with the advent of effective psychiatric medications like chlorpromazine in 1952. By around 1960, lobotomy was largely abandoned except for specific cases resistant to other therapies, and modern techniques involve much more precise incisions [2,3].

Ethical dilemmas

A severe lack of therapeutic options for the mentally ill, coupled with the alarming increase in admissions to psychiatric institutions following World War II, prompted the popularization of leucotomy despite the lack of thorough investigation. Emphasis was placed on discharging chronically hospitalized patients with psychiatric conditions as mortality rates from tuberculosis and other infections continued to rise in overcrowded institutions [1].

Lobotomy, while intended to treat severe psychoses, raised significant ethical concerns, especially when performed against a patient's wishes. This ethical dilemma is particularly challenging in cases where patients lack insight into their illness, making it difficult to determine their true preferences. Over time, public skepticism grew regarding whether the outcomes of lobotomy were worse than the conditions it aimed to address. Although lobotomized patients were often more manageable, they frequently suffered irreversible changes in personality, resulting in descriptions like "mental invalids" or "drooling zombies" [1]. Hollywood actress Frances Farmer, writer Tennessee Williams, and the sisters of American President John F. Kennedy were some of the well-known personalities who underwent this procedure.

In his 1949 paper, Jay L. Hoffman criticized lobotomy and highlighted the severe drawbacks of the procedure, noting that patients, while no longer distressed, became emotionally flat and devoid of initiative. They were often described as dull, apathetic, and lacking purpose or drive. Moniz faced criticism for downplaying the complications, inadequate documentation, and poor patient follow-up. These issues underscored the ethical complexities of using lobotomy, raising questions about whether the procedure's benefits truly outweighed its profound and often debilitating side effects [3].

The Nobel Prize awarded to Moniz has faced significant criticism. Torsten Wiesel, a fellow Nobel laureate in medicine, remarked that Moniz's 1949 prize "was a terrible mistake that caused permanent damage to thousands of patients." This sparked widespread controversy, with many questioning whether such a contentious and harmful treatment should have been honored with such a prestigious award. This led to calls for a reconsideration of Moniz's recognition [16]. However, advocates of this procedure dismissed these concerns, attributing the imperfect results to the early stages of research and viewing it as a necessary area of experimentation rather than questioning the practice itself [17].

The future of Dr. Moniz's work

Psychosurgery

The techniques developed by Dr. Moniz were brought to the United States by American Neurosurgeon Walter Freeman. He created a novel transorbital approach to decrease the invasiveness and duration of the procedure [18]. The advent of neuroleptics, beginning with chlorpromazine in 1952, quickly rendered lobotomy less relevant for treating schizophrenia, leading to a sharp decline in its use after 1960. The widespread use of chlorpromazine paved the way for the development and approval of other antipsychotic drugs, offering a much safer and more effective alternative to psychosurgery [17].

However, the field of psychosurgery was further advanced by Spiegel and Wycis in 1947 with the development of stereotactic microsurgery techniques to perform minute surgeries isolating individual bundles in the brain [19]. Further, in 1962, Foltz and White used stereotactic techniques to target the anterior cingulate gyrus to provide analgesia in a group of patients [20]. In the modern age, the use of a modified neuropsychiatry approach, which is not destructive but rather selective stimulation, is commonly used in the form of deep brain stimulation in patients with Parkinson's disease [21]. This use in Parkinson's has reignited the intrigue in the use of psychosurgical approaches in the management of other disorders such as schizophrenia and depression [22,23]. Today, procedures like bilateral anterior capsulotomy (or cingulotomy in the United States) are used primarily for chronic anxiety and OCDs that have not responded to other treatments [24,25].

Angiography

Despite advancements in noninvasive imaging techniques like computed tomography angiography and magnetic resonance (MR) angiography, cerebral angiography remains a vital diagnostic tool, with its sensitivity enhanced by modern technological developments [5]. Angiography is vital in craniotomy planning as it identifies the major arteries supplying the neoplasm, particularly in vascular meningiomas, certain gliomas, and tumors near the sella turcica. Understanding the involvement of the internal carotid artery helps prevent fatal exsanguination during surgery, thereby reducing mortality [26].

Cerebral angiography also remains essential for confirming cerebrovascular malformations, even though MR angiography is an effective screening tool. It is also indispensable for definitively diagnosing conditions such as arteritis, arterial dissection, and fibromuscular dysplasia [27]. The most common indications for cerebral angiography today are subarachnoid hemorrhage, ischemic stroke, intracerebral hemorrhage, and cerebral aneurysms [28].

## Conclusions

Once deemed groundbreaking, a medical strategy may later show unexpected negative effects. However, this does not necessarily indicate that the approach was inherently flawed or malicious. As medical knowledge advances, what was once considered effective may be reevaluated with new evidence. Initially, the strategy might have been the best option based on the available knowledge, but its limitations or side effects often become more apparent over time. This underscores the importance of continuous research and adaptation in healthcare. Egas Moniz's leucotomy, once hailed as a revolutionary treatment for severe mental illness and recognized with a Nobel Prize, illustrates this. Over time, the procedure was revealed to have significant ethical concerns and detrimental effects on patients, raising questions about whether such a controversial treatment should have been rewarded and highlighting the importance of ethics in medical advancements. Moniz's contribution to cerebral angiography, however, had a lasting positive impact on neurology by enabling more accurate diagnosis of intracranial diseases, showing that his innovations were not without enduring value.
